# The Cytotoxic Effect of Magainin II on the MDA-MB-231 and M14K Tumour Cell Lines

**DOI:** 10.1155/2013/831709

**Published:** 2013-10-09

**Authors:** Radu Anghel, Daniela Jitaru, Laurenţiu Bădescu, Magda Bădescu, Manuela Ciocoiu

**Affiliations:** ^1^University of Medicine and Pharmacy “Grigore T. Popa”, 700115 Iaşi, Romania; ^2^Laboratory of Molecular Biology, Regional Institute of Oncology Iasi, University of Medicine and Pharmacy “Grigore T. Popa”, 700115 Iaşi, Romania; ^3^Department of Cell and Molecular Biology, University of Medicine and Pharmacy “Grigore T. Popa”, 700115 Iaşi, Romania; ^4^Department of Pathophysiology, University of Medicine and Pharmacy “Grigore T. Popa”, 700115 Iaşi, Romania

## Abstract

Many studies have highlighted the tumoricidal properties of some natural peptides known to have antimicrobial virtues. Also, the increasingly higher resistance to conventional antibiotics has become a global public health issue, and the need for new antibiotics has stimulated interest in finding and synthesizing new antimicrobial peptides, which may also be used as chemotherapeutic agents. Relying on the literature, the purpose of our *in vitro* research was to assess the tumoricidal potential of magainin II on a series of tumour cell lines, namely, MDA-MB-231 (breast adenocarcinoma) and M14K (human mesothelioma). The experimental results of our study revealed that the cytotoxic effects of magainin II depend on its concentration. Its efficiency is significant at 120 **μ**M concentrations, and, although it is much lower, it persists even at 60 **μ**M concentrations. The effects were insignificant at 30 **μ**M concentrations. In our experimental research, the tumoricidal effect of magainin II was not significantly dependent on the type of tumour cell line used.

## 1. Introduction

Despite the recent progress achieved in its therapy, cancer remains a major source of morbidity and mortality worldwide. Moreover, many types of cancer, such as skin, prostate, breast, and kidney, have been on the rise. “Cancer” is actually a general term which refers to more than 100 different diseases affecting various tissues and types of cells. Nevertheless, all forms of cancer are characterized by abnormal cell proliferation caused by different inherited or environment-induced genetic mutations [1].

Although some cases of cancer may often be successfully treated by surgery and/or radiotherapy, chemotherapy is still the most commonly used treatment of advanced or metastatic cancer. However, we may confirm that, in addition to the agents already used in standard chemotherapy, finding new agents with antitumour potential is a big challenge. Many studies have focused on the tumoricidal properties of some natural peptides known to have antimicrobial virtues [2–5]. Also, the increasingly higher resistance to conventional antibiotics has become a global public health issue, and the need for new antibiotics has stimulated interest in finding and synthesizing new antimicrobial peptides, which may also be used as therapeutic agents [6–10].

Relying on the literature, the purpose of our *in vitro* research was to assess the tumoricidal potential of the cytotoxic peptide magainin II on a series of tumour cell lines, namely, MDA-MB-231 (breast adenocarcinoma) and M14K (human mesothelioma) [3, 11–13].

*Magainin II.* a cationic peptide (Gly-Ile-Gly-Lys-Phe-Leu-His-Ser-Ala-Lys-Lys-Phe-Gly-Lys-Ala-Phe-Val-Gly-Glu-Ile-Met-Asn-Ser) with an *α*-helix-like amphiphilic structure targets certain cell membranes directly, where it forms permeable ion channels that lead to depolarization and irreversible cytolysis and eventually to cell death [14, 15]. This peptide is water soluble and nonhaemolytic at its effective and amphiphilic antimicrobial concentrations. At small concentrations, it inhibits the growth of numerous species of bacteria and fungi, and it induces osmotic lysis in protozoa [7, 16].

Consequently, their amino acid composition, amphipathicity, cationic charge, and molecular size allow cytotoxic peptides to adhere to and penetrate the phospholipid bilayer of the cell membrane, thus forming trans membrane pores, which will alter cell membrane permeability and cause that cell to undergo apoptosis [2, 3, 16]. So, our experimental research was aimed at checking the hypothesis according to which the cytotoxic peptide magainin II has a tumoricidal potential of variable intensity, potential that depends both on the nature of the peptide employed and on its concentration in the living environment of the cells, as well as on the type of cell line chosen for the *in vitro *experimental study.

## 2. Materials and Methods

### 2.1. The Peptide, Cell Lines, and Cell Cultures

Magainin II, ≥97% (HPLC) is an antibiotic peptide from SIGMA Product no. M7402-1MG Lot no. 060M4823. Magainins are positively charged and amphiphilic molecules. They are known to preferentially bind to anionic phospholipids abundant in bacterial membranes with the formation of dynamic peptide-lipid supramolecular pore and cell permeabilization. Binding to artificial neutral membranes has also been demonstrated. Magainin II was freeze-dried (Sigma) in a RPMI 1640 (Sigma Aldrich) solution in a sterile environment.

According to the product specifications, this peptide is soluble in distilled water with 0.1% TFA. As the solution is meant to be inserted into cell culture medium, we have chosen RPMI as the most suitable medium. This medium also allowed us to maintain an optimum pH (7.2–7.4) as we inserted the peptide solution. The extemporaneous peptide solution was utilized immediately after preparation.

Consecutive peptide dilutions with RPMI 1640 (Sigma Aldrich) were used, whereas the working concentrations were 120 *μ*M, 60 *μ*M, 30 *μ*M, 15 *μ*M, 7.5 *μ*M, 3.5 *μ*M, 1.8 *μ*M, and 0.9 *μ*M in a final volume of 200 *μ*L/well. The peptide solution that we used and its dilutions were mixed with RPMI, and the final mixture had a pH of 7.38. Everything was treated in a sterile fashion.

**Figure 1:**
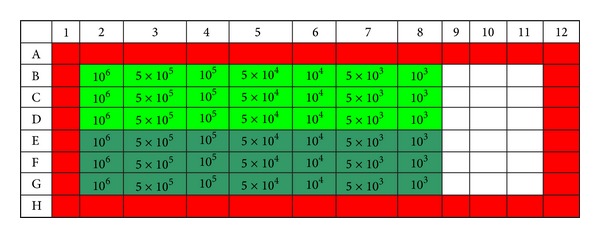
Working scheme used for tapping optimal concentration of target cells needed to assess cytotoxicity for the studied peptides. Concentrations of cells/well ranged between 10^6^ and 10^3^ target cells/well.

Cell cultures are processes by which cells are cultivated in controlled conditions. Cell lines may be grown in adherent or suspension cell cultures. In order to determine the optimal number of cells required to assess the cytotoxicity of certain natural peptides such as magainin II, we used two tumour cell lines: a line of adherent cells—M14K (human mesothelioma) and a line of suspension cells—K562 (lymphoblasts sampled from the bone marrow of chronic myeloid leukaemia patients). In our study, the optimization of the number of target cells was necessary since the peptide concentration was a variable parameter. Both cell lines were cultivated in a RPMI-1640 (Sigma Aldrich) medium with 10% SFV (*Fetal Bovine Serum*, GIBCO) [16]. Cell expansion was conducted in 250 mL flasks in order to get a sufficient number of cells (4 × 10^6^ cells for M14K and 1 × 10^7^ cells for the K562 line). The mesothelial cells were detached by trypsinization (3-minute incubation at 37°C with *Eurobio *trypsin). Only cells with viability higher than 97% were used. The actual testing was performed in plates with 96 flat-bottom wells each, using a final working volume of 200 *μ*L per well. We worked in triplicate and used two working variants; cells were incubated in simple and in 2% SFV RPMI medium. They are shown in Figure 1 coloured in light green and dark green, respectively. In order to prevent the edge effect and preserve humidity in the plate, only culture medium was pipetted in the edge columns and lines (coloured in red in Figure 1). Viability was analysed after 24 hours of incubation at 37°C, 5% CO_2_, by two methods: microscopy using the vital stain *Trypan blue* and flow cytometry using propidium iodide.

We analysed the cytotoxicity of the peptide chosen for our study in relation to two adherent tumour cell lines: MDA-MB-231 (breast adenocarcinoma) and M14K (human mesothelioma). These lines were cultivated in the same conditions as those used for the optimization of the number of target cells. The negative control was represented by target cells incubated without peptide. Viability was analysed after 72 h of incubation at 37°C, 5% CO_2_, by two methods: MTT colorimetric assay [3-(4,5-dimethylthiazol-2-yl)-2,5-diphenyltetrazolium bromide] and flow cytometry using PE Annexin V (BD Pharmingen) and 7-AAD (7-aminoactinomycin D).

*Trypan Blue Viability Assay*. Given the selective permeability of living cell membrane, trypan blue cannot be absorbed by living cells, but it can easily penetrate dead cells membranes. Consequently, when analysed with an optical microscope, dead cells are blue whereas living cells are colourless. A 1/1 cell suspension and trypan blue mixture was prepared. 10 *μ*L of this mixture was pipetted in the Bűrker-Tűrk counting chamber, and the living cells (colourless) and dead cells (blue) were counted using an optical microscope. The living cells percentage was calculated using the formula:

(1) living cell percentage (%)=living cells×100living cells+dead cells.

**Figure 2:**
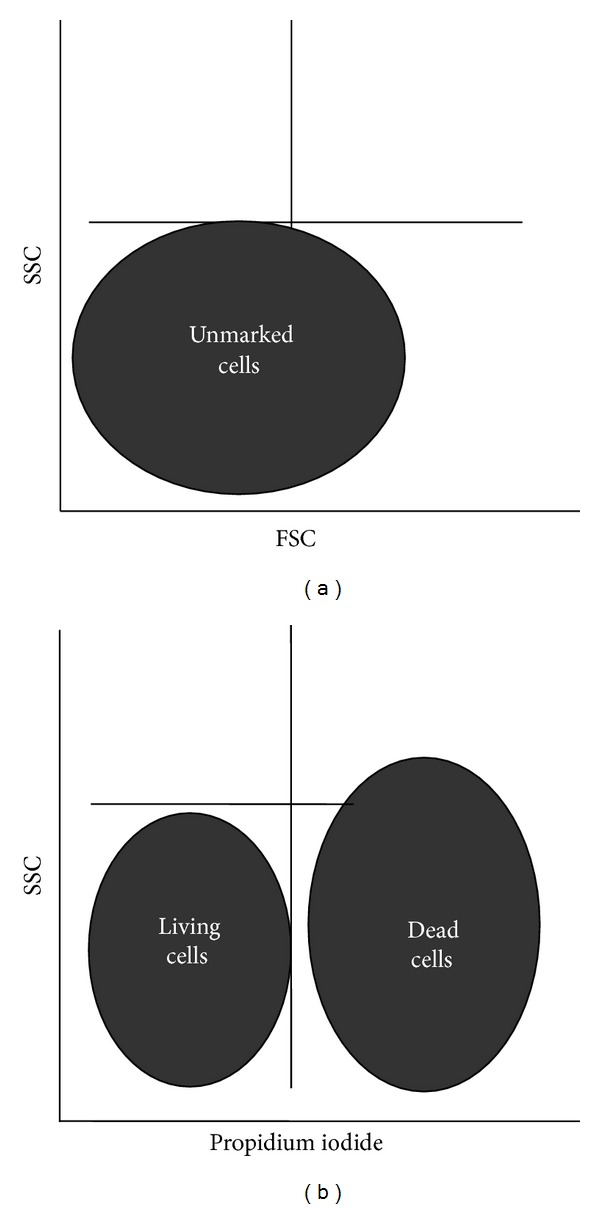
Cell arrangement according to granularity and volume (a) and fluorescence (b).

*Propidium Iodide (PI) Viability Assay*. It is used to detect and measure cell death. Propidium iodide (PI) is a vital DNA stain; this penetrates the dead cells and binds to nuclear DNA, whereas living cell membrane is not PI permeable. Propidium iodide fluorescence is read on the FL2 channel (the wavelength emitted by PI is 562–588 nm when fluorochrome excitation is 488 nm), and fluorescence intensity is directly proportional to the amount of DNA in the cells. The settings were done depending on the granularity (*SSC-side scatter*) and volume (*FSC-forward scatter*) of the unmarked cells examined with a *flow cytometer *(Figure 2).

We used a solution of 10 *μ*g/mL PI in PBS kept in darkness at 4°C. Flow cytometry labelling for cell viability used 10 *μ*L/FACS vial (200 *μ*L cell suspension, about 1 × 10^5^ cells). So, the final concentration of PI was 0.1 *μ*g/200 *μ*L cell suspension.

**Figure 3:**
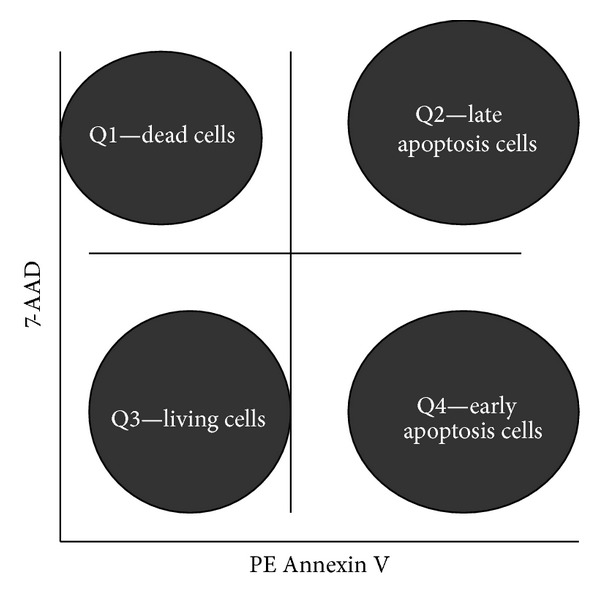
Assessment of cell viability by flow cytometry technique using the PE Annexin V and 7-AAD. Quadrant Q1 represents dead cells, Q2 represents late apoptotic cell population, Q3 represents population of living cells, and Q4 represents early apoptosis cell population.

Cell suspension was recovered in wells and distributed in FACS tubes. The adherent cells were detached by trypsination (3–10 minutes of incubation at 37°C with *Eurobio *trypsin) depending on each cell line. 10 *μ*L of PI (Consul T.S.) was pipetted in each tube after the *flow-cytometric *investigation of the unmarked cells. Data collection and interpretation were done on a FACS Canto II flow cytometer using the FACSDiva Software (Becton Dickinson) (Figure 3).

*PE Annexin V and 7-AAD (7-Aminoactinomycin D) (BD Pharmingen) Viability Assay*. This assay was used to detect and measure the apoptotic process by means of flow cytometry. Apoptosis is characterized by cell morphology changes, nuclear condensation, pyknosis, and also by a series of biochemical events causing mitochondrial membrane and nuclear DNA degradation. Phosphatidylserine, which is normally present in the inner layer of the lipid bilayer of the cell membrane, is exposed at the surface of the cells undergoing various stages of apoptosis. Annexin V has great affinity to phosphatidylserine residue, and 7-AAD was used as vital nuclear DNA stain. 96-well cell culture plates were used in our study. The first (1) and last (12) columns of wells only contained culture medium (RPMI 1640) in order to prevent the edge effect. Columns 2 and 11 of wells only contained peptide-free cell suspension (negative control). Columns 3–10 contained target cells and consecutive peptide dilutions. We worked in triplicate for the two cell lines and all the peptide concentrations we used. After 72 h of incubation at 37°C, 5% CO_2_, adherent cells were detached by trypsination (3–10 minutes of incubation at 37°C with *Eurobio *trypsin). The cell suspension was distributed in such a manner that each FACS tube had 1 × 10^5^ cells. 3 FACS tubes were assessed; the first FACS tube contained unmarked cells, the second FACS tube contained Annexin V PE, and the third FACS tube contained Annexin V PE/7-AAD. Data collection and interpretation were done on a FACS Canto II flow cytometer using the FACSDiva Software (Becton Dickinson) (Figure 3).

*[3-(4,5-Dimethylthiazol-2-yl)-2,5-diphenyltetrazolium Bromide] MTT Viability Assay*. The method is based on the ability of succinate dehydrogenase in living cell mitochondria to reduce soluble tetrazolium salts to insoluble red formazan [17]. In order to test viability by these methods, the target cells were incubated at 37°C, 5% CO_2_, for 72 h, and then 20 *μ*L of MTT (Sigma, 5 mg/mL) was added in each cell well and incubated again at 37°C for 4 more hours. The medium was then removed, and 100 *μ*L of DMSO (dimethyl sulfoxide, Roth) was added. Absorbance was measured at 570 nm against a 620 nm reference wavelength using a Tecan Sunrise plate reader. Relying on the absorbance values read in the cells incubated with the peptide studied (*A*_peptide  cells_) and depending on the absorbance value read in peptide-free cells (*A*_negative  control_), we calculated the cytostasis percentage, that is, the percentage of cells affected by apoptosis of the whole number of cells in any of the wells analysed.

(2) $$\begin{array}{c} \text{cytostasis}\;\;\%=\left(1-\frac{A_{\text{peptide}\;\text{cells}}}{A_{\text{negative}\;\text{control}}}\right)\times 100. \end{array}$$

We worked in triplicate for each cell line and for each peptide concentration. The absorbance values used to determine cytostasis were represented by the mean of the three readings for each peptide concentration and each cell line studied. 

## 3. Results

One of the first objectives of our research was to determine the optimal number of tumour cells required to be able to assess the cytotoxic potential of the natural peptide called magainin II. Such optimization was necessary since the variable parameter was represented by the consecutive peptide dilutions, and an inadequate target cell concentration would not have allowed an accurate assessment of the cytotoxic potential of the peptide included in our study. 

In the event of cell line cultivation, the initial number of cells in the culture medium is low (Figure 5(a)). Then, in adequate and controlled cultivation conditions, these cells proliferate, so that their number is significantly higher after 24 to 48 hours (Figure 5(b)). When the cell culture begins apoptosis, numerous apoptotic bodies are noted in the culture medium by microscopic observation (Figure 5(c)).

**Figure 4:**
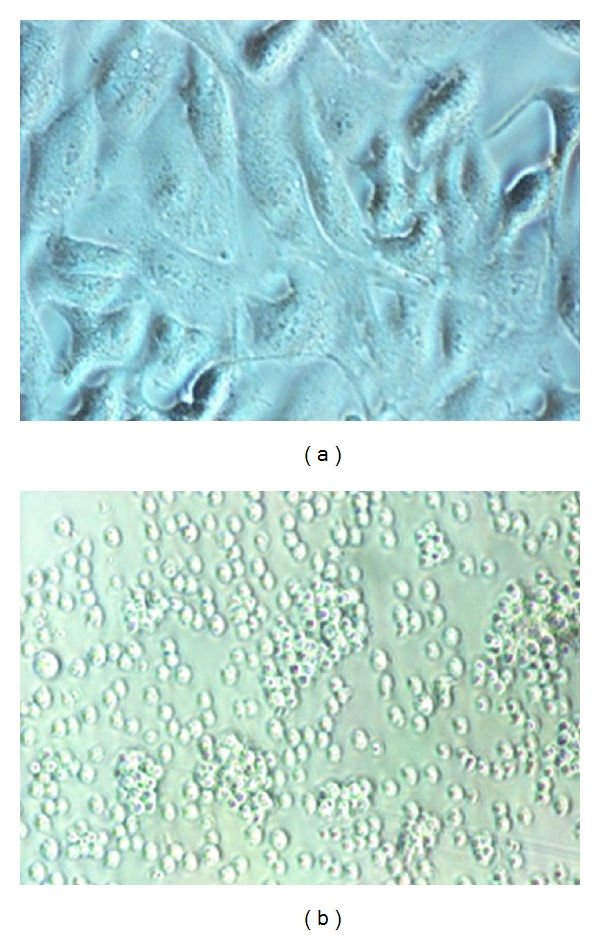
(a) Adherent cell cultures (M14K) and (b) in suspension (K562).

**Figure 5:**
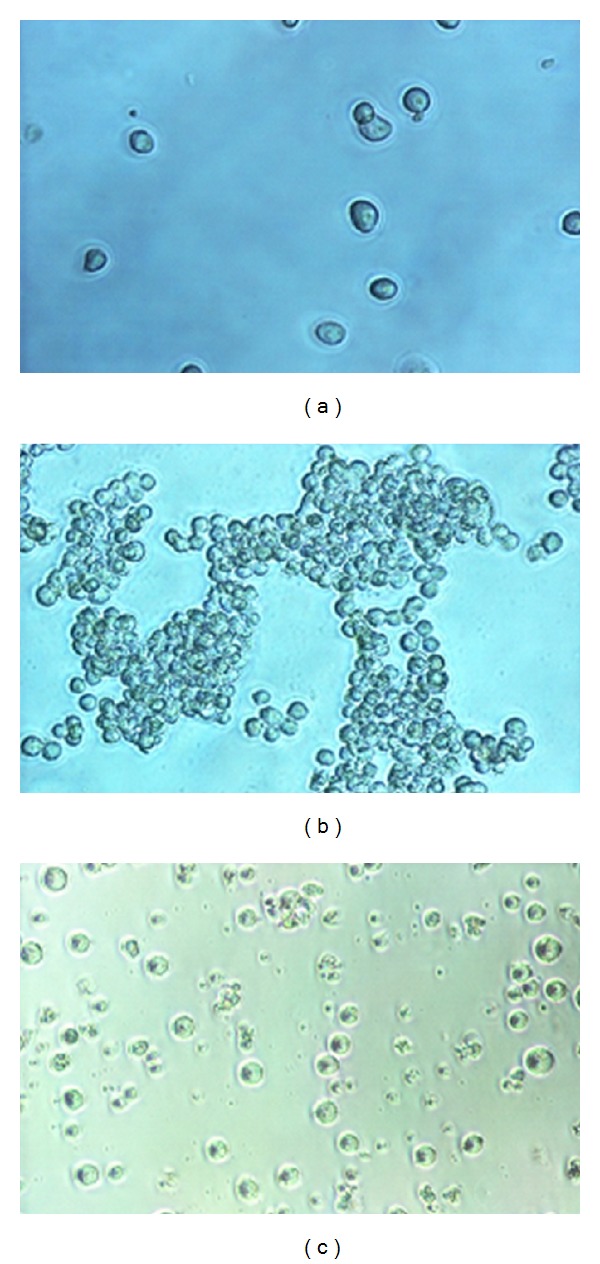
(a) Original culture in suspension cell line (K562). (b) K562 suspension culture cell line after 24 hours. (c) Apoptotic corpus in the culture medium of the K562 cell line.

Both the M14K adherent line cells (Figure 4(a)) and the K562 suspension line cells (Figure 4(b)) proliferated in 250 mL flasks in order to obtain a sufficient number of cells (4 × 10^6^ cells for M14K and 1 × 10^7^ cells for K562).

**Figure 6:**
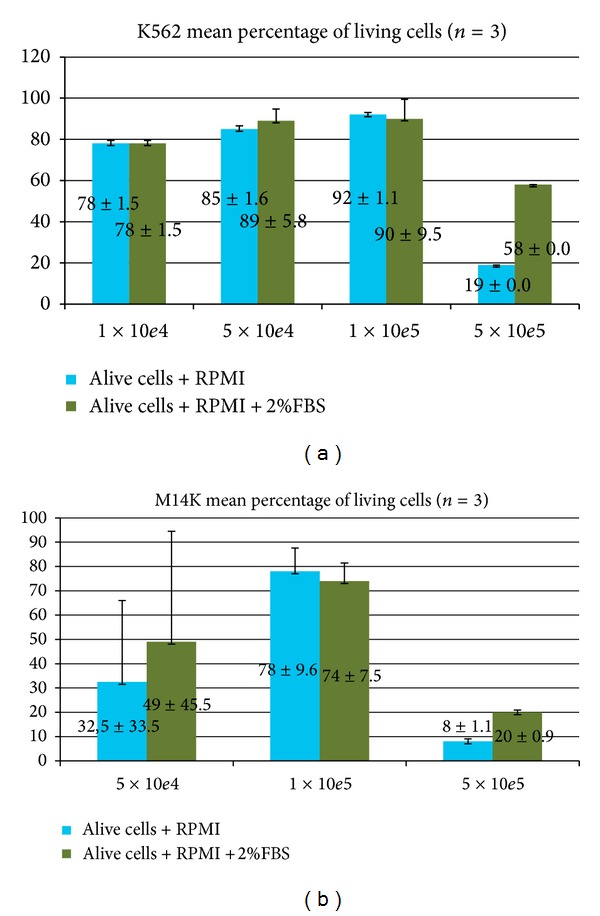
M14K and K562 24 hours incubated cell viability at 37°C, 5% CO_2_ in RPMI medium easy (blue columns) or with 2% SFV (green columns).

*Trypan Blue Viability Assay*. The *optimal viability* was 1 × 10^5^ cells/well for the two cells lines tested, which were cultivated with and without 2% SFV (Figure 6). The viability of the wells containing less than 10^4^ cells could not be determined, since less than 25 cells were detected in the counting chamber. The 10^6^ cell wells contained only dead cells, and therefore the results were not depicted in any chart.

**Figure 7:**
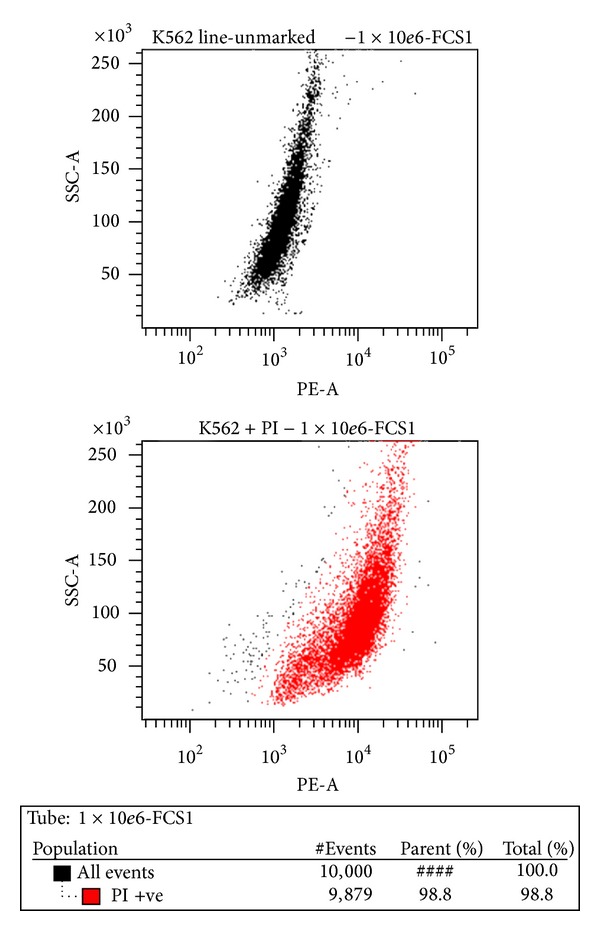
Concentration of 10^6^ cells resulted in the death of all cells after 24 hours of incubation.

*Propidium Iodide (PI) Viability Assay Results.* Flow cytometry enabled us to also determine the viability of the 10^4^ cell wells. The mortality rate in the 10^6^ cell wells was 98.8% (Figure 7). 

**Figure 8:**
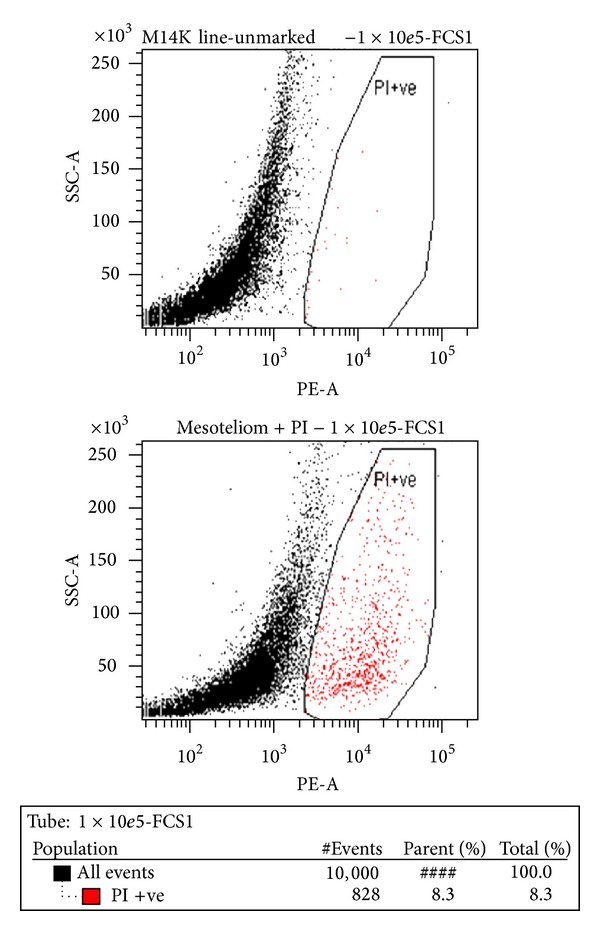
Flow cytometric identification of dead cells based on propidium iodide positivity.

**Figure 9:**
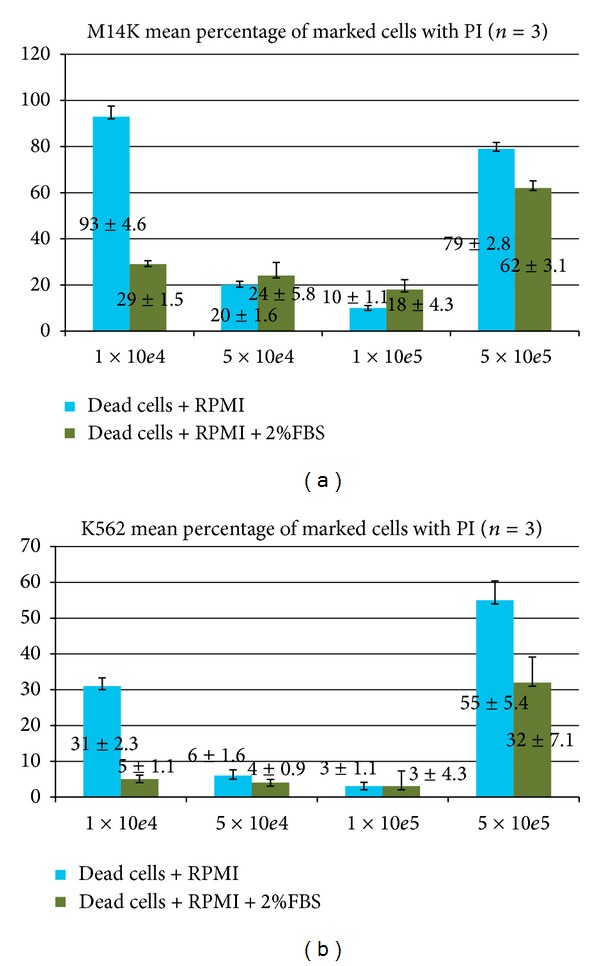
Percentage of M14K dead cells (a) and K562 dead cells (b) after incubation for 24 hours in RPMI medium easy (blue columns) or RPMI medium with 2% SFV (green columns).

Data analysis supported the findings of the trypan blue assay according to which the best working concentration was 10^5^ cells/well (Figures 8 and 9).

The experimental results we achieved allowed us to conclude that 10^5^ cells/well for 200 *μ*L of culture medium is sufficient for the performance of the proposed experiments in optimal conditions. Thus, we obtained a useful *in vitro* experimental model designed to monitor the cytotoxic potential of the natural peptide called magainin II. 

In addition to this *in vitro* experimental model, another objective of this study was to assess the cytotoxic potential of magainin II on the MDA-MB-231 and M14K tumour cell lines. MDA-MB-231 is an adherent breast adenocarcinoma cell line, one of the three known breast cancer cell lines: MCF-7, MDA-MB-231, and T-47D [18, 19]. The literature has proven that magainin II has a cytotoxic effect on the MCF7 tumour cell line at concentrations that exceed 200 *μ*g/mL (>80 *μ*M) [13]. The purpose of our study was to determine whether magainin II has the same cytotoxic effect on the MDA-MB-231 cell line.

The MTT viability assay revealed that the cytostasis of 120 *μ*M of magainin II on the MDA-MB-231 tumour cell line exceeded 50%. The cytostasis persisted even at 30 *μ*M concentrations, but it was absent at 15 *μ*M concentrations (Table 1). 

The absorbance values considered for cytostasis determination were represented by the mean of the three readings of each peptide concentration and each cell line analysed. There were no statistically significant differences between the three absorbance readings. 

Flow cytometry allowed apoptosis assessment for the MDA-MB-231 cell line incubated with the three peptides. 5000 events/well were acquired, and the results were compared with the viability of the peptide-free cells (negative control).

**Figure 10:**
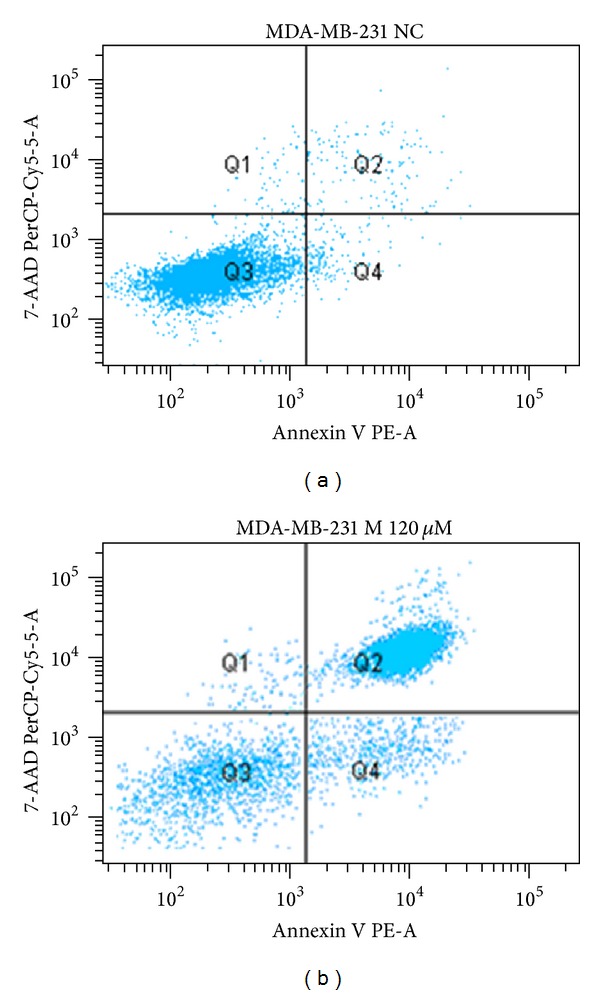
Distribution of cell populations based on fluorescence intensity of 7-AAD markings and V Anexin MBA-MB-231 cells in the absence (CN) and in the presence of cytotoxic peptide at a concentration of 120**** *μ*M (M120) after incubation for 72 hours in RPMI.

**Figure 11:**
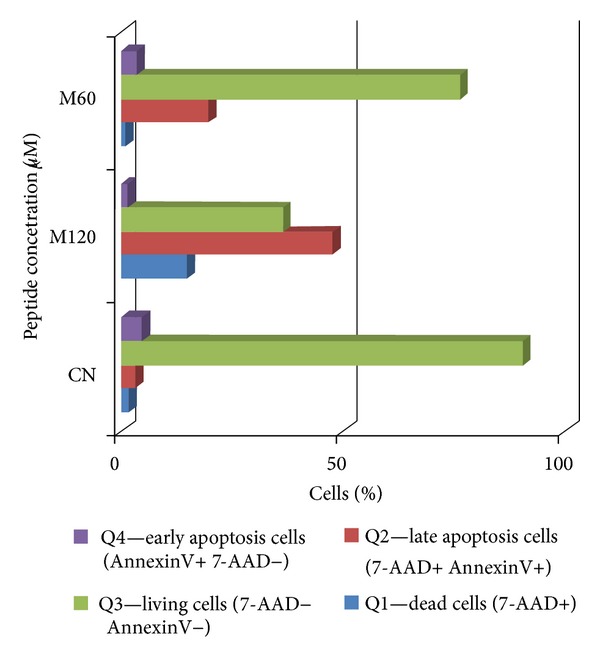
Assessment of apoptosis in MDA-MB-231 absence and presence of cytotoxic studied peptides: magainin II (M) at concentrations of 120 *μ*M and 60 *μ*M after incubation for 72 hours in RPMI.

The results obtained by this technique were confirmed by the MTT assay; that is, about 50% of the cells incubated with magainin II (120 *μ*M) underwent apoptosis (1.4% are dead cells—quadrant Q1, 14.8% in early apoptosis—quadrant Q4 population, and 47.% in late apoptosis—quadrant Q2 population) (Figures 10 and 11, Table 2). 

*M14K Is an Adherent Human Mesothelioma Cell Line.* Mesothelioma is a very aggressive malignant tumour, which occurs not only on surfaces covered with mesothelial cells, most commonly in pleural cavities, but also in the peritoneum, pericardium, and soft paratesticular tissue. As its metastasis potential amounts to 75% of the patients and its response rate to various chemotherapeutic agents is below 20%, mesothelial cells are an adequate target for the assessment of new chemotherapeutic agents [20, 21]. Among the 3 histological subtypes of mesothelioma, our experiment used the M14K mesothelial epithelial cell line.

The evolution of the M14K cells after 72 h of incubation with magainin II was similar to that of the MDA-MB-231 cells, meaning that cytostasis exceeded 50% when a 120 *μ*M concentration of magainin II was used (Table 3). Magainin cytotoxicity was significantly lower at 30 *μ*M concentrations and absent at 15 *μ*M concentrations in both tumour cell lines (Tables 1 and 3).

Therefore, when incubated with magainin II, cell viability was equally low in the MDA-MB-231 tumour cell line and in the M14K tumour cell line. Our experimental research revealed no considerably different behaviour of the various types of tumour cells under the action of the same concentration of cytotoxic peptide.

**Figure 12:**
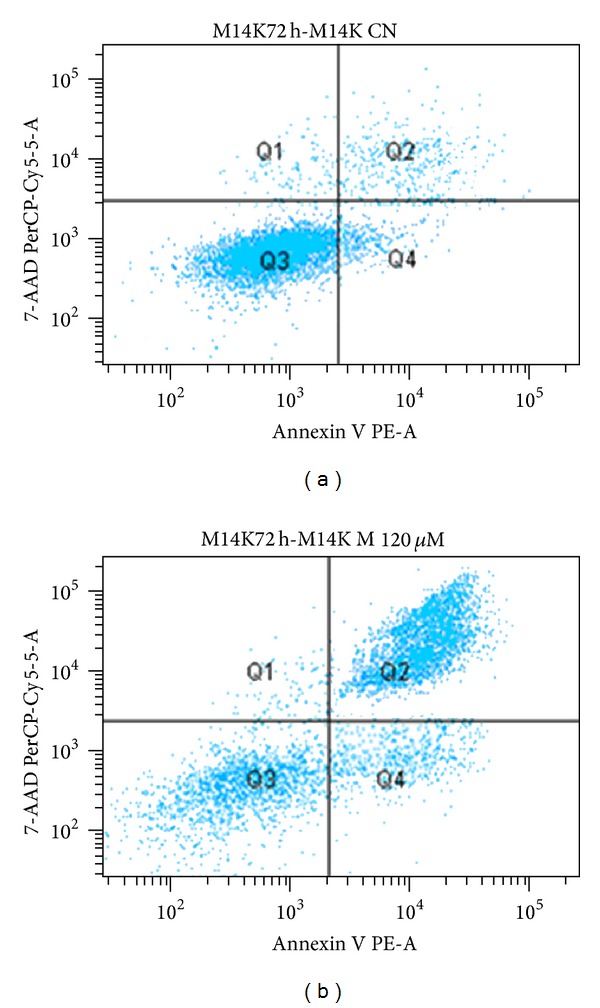
Distribution of fluorescence intensity of 7-AAD markings and Annexin V M14K cells in the absence and in the presence of cytotoxic peptide after incubation for 72 hours in RPMI.

**Figure 13:**
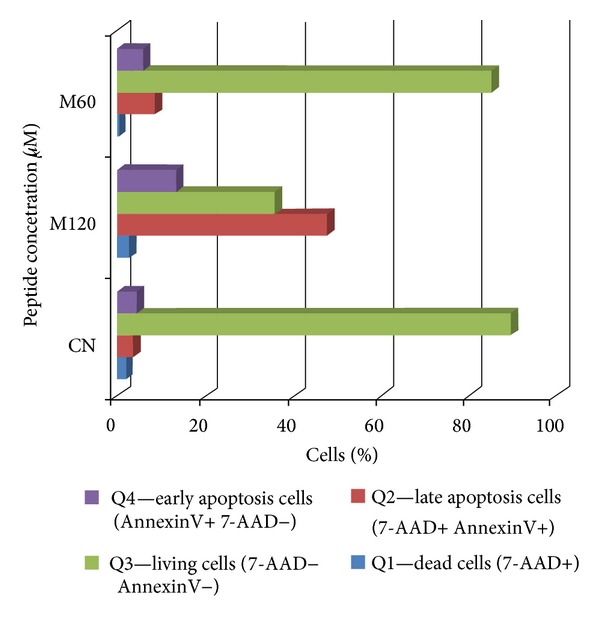
Assessment of apoptosis in MDA-MB-231 absence or presence of cytotoxic studied peptides: magainin II (M) concentration of 120 *μ*M and 60 *μ*M after incubation for 72 hours in RPMI.

The results obtained by flow cytometry that assessed the apoptosis of the M14K line cells incubated for 72 h with different concentrations of magainin II according to the method described above were confirmed by the MTT technique (Figures 12 and 13, Table 4). We found that over 50% of the cell population underwent apoptosis (47.8% late apoptosis—quadrant Q2, 13.5% early apoptosis—quadrant Q4) as compared to the negative control which had a cell viability of only 89.6% (quadrant Q3) (Figure 13, Table 4).

## 4. Discussions

The activity and specificity of antimicrobial peptides rely on *primary sequence* (primary structure of the amino acid chain); *net electric charge* (the value which depends of the medium pH); *secondary structure*—most antimicrobial peptides do not have a well-defined secondary structure in aqueous solutions; yet, at the cell membrane level, they may include a wide range of secondary structures such as *α*-helix (the most frequent in the environment), *β*-sheet, or cyclic structures; *hydrophobicity*—the percentage of hydrophobic amino acid residue in the primary peptide sequence, which is generally ~50% for antimicrobial peptides; *amphipathicity*—the property of antimicrobial peptides consisting of a series of hydrophilic and hydrophobic amino acids on both sides of the *α*-helix structure; and *polar angle*—a measure of the relative proportion of the polar part of a *α*-helix structure, as compared to the hydrophobic part [2–4, 10, 22]. Electrophysiology and molecular biology techniques have shown that peptide structures can alter the cell membrane by (1) interactions with intrinsic ion carrier proteins and/or (2) ion channel formation [16, 22, 23]. These two mechanisms of action, which are not mutually exclusive, can cause changes promoting abnormal electric activity increase, distorted signal transduction and can eventually lead to cell death [16, 23, 24].

As the molecular protein-membrane interaction mechanisms make up an important border of cell biology, further investigations on these peptides may provide useful information on the small peptide-cell membrane interaction mechanism, as well as on the manners in which this process may be modulated. Although the precise mechanism of antimicrobial peptide action is still controversial, specialists agree on one thing, namely, that these peptides distort cell membranes selectively and that the structural amphipathic peptide layout is thought to play an important role in this mechanism [2, 25–27].

New information on the peptide binding to cell membrane properties, which are the grounds of intracellular signalling, is a fundamental contribution to this cytotoxic peptide research field [28, 29]. Recent studies have shown that solid phase RMN spectroscopy is a unique method of studying noncrystalline insoluble complexes formed between plasma membrane lipids and these antimicrobial peptides [30, 31].

The antimicrobial action of peptides involves several stages such as cell membrane adsorption, aggregation at the aqueous medium/lipid bilayer interface and peptide insertion into the bilayer, and membrane pore formation [32]. Among the models used to explain the mechanism of the cell membrane distortion process under the action of cytotoxic peptides, we should mention the *carpet *model or classical pore model and the *toroidal pore* model [32]. Membrane destabilization by the mechanisms described above may be followed by a process of translocation of certain peptides on the inner side of the cell membrane, which allows them to interact with intracellular targets and alter certain processes at this level [32]. 

Considering all these issues tackled by the literature, our experimental research analyses the assumption according to which the peptide called magainin II has a tumoricidal potential and attempts to establish whether the intensity of the effect is dependent on the nature of the peptide used and on its concentration in the living cell environment, as well as on the type of cell line chosen for the *in vitro *experimental study. In order to underline these phenomena, we reckoned that 10^5^ cells/well in a final culture environment volume of 200 *μ*L would be the optimal cell concentration necessary to assess the cytotoxic potential of the peptides.

The objectives of our research were fulfilled by the assessment, by means of the MTT technique, of the *in vitro* cytotoxic effect determining the cytostasis process in various types of cells (MDA-MB-231 and M14K cell lines), depending on the type and concentrations of cytotoxic peptide used. This cell viability monitoring technique in the presence of a supposedly toxic compound was confirmed by the flow cytometry technique using the 7-AAD and PE Annexin V fluorochromes. This technique was also employed to determine the apoptosis stage of the tumour cells after 72 hours of incubation in the presence of the peptide. The results obtained were also compared with the negative control (tumour cells incubated in the absence of peptide).

## 5. Conclusions

In our research, the cytotoxic effects of magainin II depended especially on the concentration of the peptide in the living environment of the tumour cell, being significant at 120 *μ*M concentrations and persisting even at 60 *μ*M concentrations. These effects were insignificant at 30 *μ*M concentrations. Their tumoricidal effect was not significantly influenced by the type of tumour cell line under survey.

We hope that our experimental data will be followed by *in vivo* testing of the tumoricidal potential of these peptides. The ultimate goal would be the discovery of agents with efficient antitumor properties, that is, with maximum tumoricidal effect and minimum toxic side effects.

The originality of our research consists, on one hand, of the fact that there are not many studies dealing with the tumoricidal properties of the cytotoxic peptides referred to and, on the other hand, on the need to research and develop new more efficient and less toxic therapeutic variants of tumour cell destruction as compared to the currently used cytostatic drugs.

## Figures and Tables

**Table 1:** The percentage of citostasis (the stop of the cell's proliferation and development until the cell death) was calculated as: (1−*A*) × 100 = % citostasis, where *A* = the ratio between cells' absorbance treated with peptide and absorbance of untreated cells. In this table we presented the results for the citostatsis of the human mammary adenocarcinoma line cells incubated for 72 hours with magainin II in RPMI.

Tumour cell lines MDA-MB-231 cytostasis % (MTT test)	
Peptide concentration	Magainin II
120 *μ*M	59.44
60 *μ*M	23.54521
30 *μ*M	13.6974
15 *μ*M	−19.9642
7,5 *μ*M	−50.761
3,5 *μ*M	−59.9821
1,8 *μ*M	−62.6679
0,9 *μ*M	−64.906

**Table 2:** The percentages of living, apoptotic, and dead mammary adenocarcinoma cells were determined by flow cytometry, as shown in this table, where Q1 quadrant includes dead cells (7-AAD positive), Q2 quadrant contain late apoptotic (7-AAD positive, Annexin V positive), and Q4 quadrant contains early apoptotic cells (Annexin V positive, 7-AAD negative). Viable cells (7-AAD negative, Annexin V negative) were enclosed in the Q3 quadrant.

MDA-MB231 5000 events/well	Q1—dead cells (7-AAD+)	Q2—late apoptosis cells (7-AAD+ Annexin V+)	Q3—living cells (7-AAD− Annexin V−)	Q4—early apoptosis cells ( Annexin V+ 7-AAD−)
	%	%	%	%
Negative control (NC)	1.8	3	90.5	4.7
Magainin II 120 *μ*M (M120)	1.4	47.5	36.3	14.8
Magainin II 60 *μ*M (M60)	0.8	19.6	76.2	3.4

**Table 3:** The results for the citostatsis (%) of the human mesothelioma line cells incubated for 72 hours with magainin II in RPMI.

M14K tumour cell lines cytostasis % (MTT test)	
Peptide conc.	Magainin II
120 *μ*M	53.95
60 *μ*M	13.37522
30 *μ*M	4.48833
15 *μ*M	−3.68043
7,5 *μ*M	−18.5817
3,5 *μ*M	−22.8905
1,8 *μ*M	−48.5637
0,9 *μ*M	−63.2855

**Table 4:** The percentages of living, apoptotic, and dead mesothelioma cells were determined by flow cytometry, as shown in this table, where Q1 quadrant includes dead cells (7-AAD positive), Q2 quadrant contains late apoptotic (7-AAD positive, AnnexinV positive), and Q4 quadrant contains early apoptotic cells (AnnexinV positive, 7-AAD negative). Viable cells (7-AAD negative, AnnexinV negative) were enclosed in the Q3 quadrant.

M14K 5000 events/well	Q1 (7-AAD+)	Q2 (7-AAD+ Annexin V+)	Q3 (7-AAD− Annexin V−)	Q4 (Annexin V+ 7AAD−)
	%	%	%	%
Negative control (NC)	2.1	3.8	89.6	4.5
Magainin II 120 *μ*M (M120)	2.8	47.8	35.9	13.5
Magainin II 60 *μ*M (M60)	0.3	8.6	85.2	5.9
